# Family-Centered Care in Adolescent Intensive Outpatient Mental Health Treatment in the United States: A Case Study

**DOI:** 10.3390/healthcare13091079

**Published:** 2025-05-06

**Authors:** Henry W. Kietzman, Willem L. Styles, Liese Franklin-Zitzkat, Maria Del Vecchio Valerian, Eunice Y. Yuen

**Affiliations:** 1 Department of Psychiatry, Yale University, New Haven, CT 06511, USA; henry.kietzman@yale.edu (H.W.K.); liese.franklin-zitzkat@yale.edu (L.F.-Z.); 2 Yale New Haven Hospital, New Haven, CT 06511, USA; willem.styles@ynhh.org (W.L.S.); maria.delvecchiovalerian@ynhh.org (M.D.V.V.); 3 Yale Child Study Center, New Haven, CT 06511, USA

**Keywords:** family-centered care, adolescent mental health, intensive outpatient program

## Abstract

Background: Social isolation, national turmoil, and an adolescent mental health crisis in the wake of the COVID-19 pandemic have resulted in a significant uptick in inpatient admissions and re-admissions for high-risk patients. This trend persists even as the pandemic wanes. Intensive outpatient programs (IOPs) serve as a critical steppingstone between the community and inpatient mental health services, providing comprehensive psychiatric care for at-risk youth. Significant research has identified family engagement as a key element of successful collaborative care in adolescents. Objectives: This article provides models of family-centered care in the adolescent IOP through a case study detailing the six-week course of care of an adolescent struggling with increased emotionality and distress intolerance in the context of family conflicts. Methods: This case highlights five family engagement components, including (1) family-centered psychiatric medication management, (2) individualized case management, parental education, and peer support, (3) Measurement Based Care (MBC) family assessment and feedback sessions, (4) Dialectical Behavior Therapy (DBT) multi-family skill groups, and (5) Compassionate Home Action Together (*CHATogether*) family intervention to address teen–parent relational health and communication. Results: This case showed improvement in depressive and anxiety symptoms, family conflict behaviors, self-reported suicide risk, and help-seeking attitudes towards parents/adults. The case family, along with others (n = 26), endorsed the parent peer support groups’ acceptability and feasibility implemented in the adolescent IOP. Conclusions: This article emphasizes the importance of family engagement during clinical care and provides a practical guide to implement collaborative family-centered therapeutic interventions in intensive outpatient services.

## 1. Introduction

Families affect the overall health of adolescents and vice versa. This bidirectional influence is particularly crucial when the adolescent is struggling [1]. Since the COVID-19 pandemic, mental health challenges in adolescents, including suicidal and non-suicidal self-injurious behaviors, have increased dramatically [2]. These degradations in adolescent mental health may be attributable, in part, to relative isolation [3] and family chaos [4]. The unrepaired damages to teens and their families have continued as the pandemic wanes, as demonstrated by a persistent increase in behavioral health service utilization [5] and poorer overall mental health and well-being [6,7]. Therefore, involvement of the family in the adolescent’s mental health treatment, also known as family or parental engagement, ensures the greatest possible treatment outcomes [1]. Such family engagement is not simply the act of participation, but a major factor enhancing the attendance rate to services and adherence to suggested treatments [8].

Several hospitals in the U.S. and abroad have adopted family-based therapy models into their intensive outpatient programs (IOPs) or partial hospitalization programs with promising outcomes. For example, the Family-Based Integrated Care (FBIC) model at the Alpert School of Medicine at Brown University encourages examination of both cognitive and affective consequences of family dynamics in supervised sessions at their partial hospitalization programs in 1998 [9,10]. Similarly, in 2006, a modified version of the Penn Resiliency Program was developed [11], which includes a parental module that provides a manualized, psychoeducational group approach to teach parents skills comparable to those being learned by their children, with an emphasis on teaching parents how to use these skills while parenting [12].

**Figure 1:**
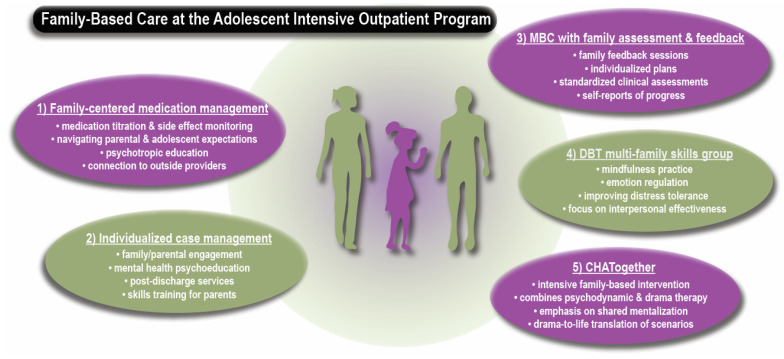
Schematic framework showing 5 family engagement components in the adolescent IOP. (1) Family-centered medication management and psychoeducation, (2) individualized case management, parental education, and peer support, (3) measurement-based care (MBC): family assessment and feedback sessions, (4) Dialectical Behavior Therapy (DBT) with multi-family skill groups, and (5) Compassionate Home, Action Together (*CHATogether*), an individualized family intervention which focuses on conflict resolution and cohesion using psychotherapeutic approaches of psychodynamic psychotherapy, drama therapy, and Cognitive Behavior Therapy [15,16].

IOPs provide a cost-effective model of care that can decrease hospital readmissions with proper post-discharge planning [13]. Adolescents and young adults can receive 15 h per week of treatments in a supportive environment. IOPs further permit continued school or work attendance, promoting the development of appropriate social networks and avoiding out-of-home placement [14]. It is also an ideal starting point to introduce family-based interventions. As schematized in Figure 1, a representative case illustrates how different family-centered care approaches can be effective to help adolescents and their families.

## 2. Data Collection

Institutional review board approval from the Yale Human Investigations Committee was obtained (#2000034837). Written informed consent was obtained from the patient and the family for the study and the publication. The case was de-identified with modifications to protect the family’s privacy. The case was an existing patient enrolled in Yale New Haven Hospital Adolescent Outpatient Behavioral Services. The patient attended the 6-week DBT IOP track, which consisted of 3 h per day, 4 days per week of group therapy, weekly medication management, and case management. In addition to these services, the patient and her parents received family-centered care, as shown in Figure 1.

The inclusion criteria at IOP included patients who required a higher level of care from the conventional outpatient psychiatric settings. The case study reported the patient with significant family relational conflicts, regardless of the official diagnosis. IOP excluded patients with high acuity psychiatric conditions including substance intoxication or withdrawal, active psychosis, eating disorder, or severe cognitive impairment.

Admission assessments included PHQ-9 modified for teens [17], Behavior Assessment System for Children 3rd Edition (BASC-3) [18], and Life Problems Inventory, Emotion Dysregulation Scale (LPI) [19,20]. Using a 5-point Likert scale (“strongly agreed” to “strongly disagreed”) delivered by the HIPAA-secured Yale Qualtrics system, we evaluated the parent peer support group satisfactions. Other validated measurements were collected at IOP admission, and discharge included Patient-Reported Outcomes Measurement Information System (PROMIS) depression and anxiety symptoms scales [21], Conflict Behavioral Questionnaires (CBQ) [22], 9-item Concise Health Risk Tracking-Self-Report (CHRT-SR-9) [23], and seeking adult help to discuss suicide concerns [24].

## 3. Isabella’s Case

As shown in Table 1, Isabella is a 15-year-old girl with major depressive disorder (MDD) who presented to IOP following her first psychiatric hospitalization for a Tylenol overdose after an argument with her parents for making an inappropriate *TikTok* video. The video led to derogatory comments from her peers, bullying in school, and harassment on social media. Emotionally distraught, she quickly turned to impulsive acts, such as yelling, self-harm, and running away.

Following the argument with her parents about the *TikTok* video, Isabella became agitated and screamed “I hate you.” She ran to the medicine cabinet and swallowed 15 Tylenol pills in front of her parents. Isabella was then taken to the emergency room and admitted to the inpatient psychiatric unit. Her parents criticized her overdose attempt as “wrong” and “manipulative”, stating “she is so selfish, and never knows how to take responsibility of her own life”.

Isabella’s parents each had different parenting styles and often argued in front of Isabella and her siblings. Both parents immigrated from Puerto Rico (PR) before their children were born. Isabella’s father, who was often busy at work, had very little time at home. Thus, most of the parenting roles were left to Isabella’s mother. The mother had significant childhood trauma and grew up with violence in her family and neighborhood in PR. Both parents have little trust in the U.S. medical system. Isabella subsequently experienced quite authoritarian parenting with little warmth in parental communication. Her parents had previous child protective services involvement due to emotional abuse towards Isabella.

A PHQ-9 modified for teens was used to confirm Isabella’s MDD diagnosis. Isabella scored 16 and was determined to have moderately severe depression (Table 1) [17]. Isabella continued her inpatient medications including Abilify 5 mg and Intuniv 2 mg to target her emotional reactivity and impulsive behaviors.

Towards the end of IOP treatment, Isabella and her parents were able to develop new coping and compassionate communication even when Isabella was frustrated. The family has now started to enjoy family cooking activities together. Parents received their own individual therapy, which they found helpful in their co-parenting. The family continued to receive family therapy at the outpatient clinic.

## 4. Family-Centered Medication Management

In the age of the internet, social media, and artificial intelligence, it can be stressful for parents to consent the use of medication for their children. Parents frequently face overwhelming information accompanied with fear of side effects or associated stigma [25]. In some cases of minority families, cultural beliefs and past experience of systematic mistreatment or lack of access to care can affect the parental attitude toward youth receiving proper medications [26]. There is also a rising trend of self-diagnosis and self-guided treatment using unreliable resources. To encourage adolescents’ medication adherence without going astray, there is a critical need of family collaboration and support for administering and storage [27].

As medication non-adherence is one of the strongest predictors of psychiatric readmission [28], the need for timely and considerate psychiatric aftercare is essential. Evidence suggests that time spent in medication management is effective, as the risk of re-hospitalization was 10% lower for each additional hour of outpatient therapy and medication support adolescents received within the first six months post-discharge [29]. IOP settings can address this need for patients coming from inpatient psychiatric hospitals [28], particularly through building a therapeutic alliance with the whole family. The families then have the opportunity for regular individualized counseling with the psychiatrist, addressing existing concerns, and appropriate psychoeducation on diagnoses and medications.

## 5. Family Engagement in Isabella’s Medication Management and Outpatient Service Connection

During Isabella’s treatment at IOP, she met weekly with her psychiatrist. Isabella noted that she had difficulty falling asleep prior to her hospitalization, but her medications were helping her get restorative sleep. Side effects of her medications including dizziness, sedation, muscle stiffness, or increased suicidality were closely monitored through the collaborative efforts among IOP staff, psychiatrist, and Isabella’s family. To ensure medication compliance, Isabella’s psychiatrist had weekly family phone sessions to answer any medication questions and to co-develop plans for medication administration, storage, and safety. Isabella’s mother was concerned about her reactivity in response to distress but stated that overall, there was a large improvement in her symptoms. To target her depression, the psychiatrist guided a shared discussion with Isabella and her parents about the pros and cons of starting an anti-depressant medication. However, after some thoughtful consideration, Isabella and her family preferred to stay with the medication regimen she was prescribed at the hospital and addressed her depressive symptoms through other non-pharmacological means. Moreover, Isabella discussed with her psychiatrist about her family’s relational stress. The psychiatrist further provided psychoeducation to the parents that such family stressors can be detrimental to Isabella’s mental health, particularly when a child overhears parental arguments. Her psychiatrist thus provided an additional touchpoint for supportive psychotherapeutic intervention beyond the medication management. After Isabella’s six week stay at IOP concluded, her psychiatrist connected her with an outpatient psychiatrist to continue her care.

In medication management, the psychiatrist is in ongoing contact with the adolescent and parents to address concerns on symptoms uncertainty and medication side effects. This is critical to maintain positive attitudes towards care, inspire patient insight, and build a relationship with the family—key factors predicting medication adherence [27]. Many adolescents and their parents perceive the potential benefits of medication differently [30]. Parents may see medications as a fix to the problem with biological means instead of considering other communicative and behavioral factors [26]. As adolescents transition towards more independence from their parents, they may resist the opinion of their parents on their medication. This discrepancy between family members can perpetuate an adolescent’s experienced stigma toward taking medications and create stress that decreases adherence [31]. Furthermore, cultural factors play into medication adherence, with U.S. Latine populations having higher rates of medication non-adherence, particularly if monolingual Spanish speakers are from a lower socioeconomic status [26,32]. Similarly, adherence among Asian Americans is frequently lower than other ethnicities, perhaps due to the prioritization of the family unit and the stigma of potentially shaming the family with a mental health diagnosis [33]. Thus, it is the nuanced role of the psychiatrist to support family cohesion and engagement—with an understanding of each family’s unique cultural lens—that can serve as a protective barrier against stigma towards pharmacological interventions [26].

## 6. Individualized Case Management, Parental Education, and Peer Support Program

Parents are stressed by increasingly more complex and difficult roles since the pandemic [34], commonly feeling incompetence, guilt, or shame regarding their children’s mental health struggles. Clinicians provide individualized case management that eases logistical barriers and strengthens parental well-being. Appointment reminders, clear treatment expectations, and status updates on their child’s progress can systematically reduce the mental burden for parents with busy schedules [8]. Case management conversations between clinicians and family members serve as an opportunity to identify needs and to foster parents’ ability to model the use of coping skills for their adolescents. Effective case management also builds parental perceptions that the benefits of receiving mental health treatment outweigh the costs through psychoeducation and problem-solving barriers, a key predictor in continued family engagement in treatment [8].

The IOP offers psychoeducation and skills training tailored for caregivers. Table 2 shows topics include mindfulness, anger management, crisis response, understanding psychiatric diagnosis and medications, and motivational interviewing skills such as Open questions, Affirmations, Reflective listening, and Summary reflections (OARS) [35]. The parent peer support groups also offer parents a moment to share advice and support with other parents. This encourages parents to shift their perspectives on mental health, connect to others, and participate in behavioral change at home by modifying their own behaviors and acquiring new behaviors to demonstrate to their adolescents.

## 7. The Impacts of Case Management, Education, and Peer Support for Isabella’s Parents

Isabella’s clinician reached out to her parents throughout the course of treatment (at least once a week but more frequently when situations required more urgent parental attention). As the case manager, the clinician assisted the parents in problem solving logistic issues, ensuring Isabella could attend treatment. When Isabella’s parents grew concerned about the impact of Isabella’s mental health on her grades, the clinician assisted them in communicating with school administrators. Clear communication helped minimize the parental perceptions that therapy attendance was interfering with progress and instead re-centered the focus on ongoing therapeutic benefits for Isabella.

Isabella’s clinician provided psychoeducation to the parents on her mental health diagnosis. Her parents were coached on coping skills that they could model when Isabella experienced emotional dysregulation at home. Together, the family discussed ways to make the home safer for Isabella in the event of a crisis, such as removing her access to over-the-counter medications, and methods of demonstrating to Isabella that her parents were listening empathetically when Isabella expressed her feelings. Isabella’s parents also attended the weekly peer support program, allowing for important connection with other caregivers, vital during the isolation often felt when supporting an adolescent through a mental health crisis.

Gradually, Isabella’s parents began to see her symptoms as not “just bad behaviors” but instead as challenges that she was working to overcome. Isabella’s clinician explained how group and individual therapy sessions helped Isabella with emotional regulation, focusing on changing her patterns of thinking and behaving. Isabella’s parents began to see therapy as a valuable tool in helping her to thrive. As they were continuously included in discussions on Isabella’s progress, allowing for a parent-clinician alignment with Isabella’s treatment. The new skills and attitudes were further reinforced when Isabella’s parents attended the parent peer support program. Isabella’s parents learned helpful ways to respond to Isabella’s mental health concerns while feeling validated by other parents. As a result, when Isabella became distressed at home, her parents responded using advice given to them by other parents, as well as their child’s clinician. Isabella’s parents also coached her in mindfulness techniques they learned, such as guided breathing and meditation. As shown in Table 3, Isabella’s parents, along with 96–100% of other families, strongly agreed or agreed that parent peer support group was acceptable and feasible to participate as part of the IOP treatment. Parents also provided feedback on topic of their interests, such as managing teens with substance use and online self-diagnosis behaviors.

There are many barriers to family engagement in adolescent mental health services. Family characteristics, such as marginalized racial/ethnic minority with language barriers, low socioeconomic status with limited social support, single-parent household and parental mental illness, predict a lower rate of family engagement in youth mental health treatment [1]. Some families may resist programs due to culture and stigma. Miscommunication or the lack of a therapeutic alliance can lead families to believe a program cannot address their needs, or that the providers in the program are lacking empathy and are judgmental.

Treating adolescents as individuals minimizes the daunting approach of treating the family as a whole. However, in-depth family engagement often bears fruit by identifying root causes of concerns, encouraging long-term collaboration, and promoting resilience [36]. The principal elements of family support needs can be categorized as instructional, informational, advocacy, and emotional support [37]. Instructional support builds parents’ skills managing the family as well as their own emotional wellbeing. Informational support provides education for parents to understand their teen’s development, the impacts of mental illness, treatment options, and resources available in the community. Advocacy support empowers parents by providing resources, training, and understanding of their rights as parents for their youth. Emotional support provides social connection among parents sharing similar identities as caregivers for youth with mental illness. Interestingly, clinician-led family support programs emphasizing instructional and informational components produce better family engagement in treatment [10].

## 8. Measurement-Based Care: Family Assessment and Feedback Session

Mental health recovery for adolescents can be a winding journey that needs guidance, perseverance, and support. Measurement-based care (MBC) [38] can serve as a lamppost illuminating the way. Its individualized process has three main components–*Collect*, *Share*, and *Act*. In the 6-week IOP structure, data of validated assessments can be collected from both adolescents and their parents. Feedback from families along with data collected are invaluable for treatment direction [39]. This information not only helps make appropriate adjustments during the treatment but is also essential to keep parents engaged with the family-centered discharge plans.

The assessment results and summaries of family feedback sessions (FFS) with the adolescents and their parents are entered into the hospital’s electronic medical record system and patient portals. This provides accessibility for both clinicians and families. Assessments in adolescent IOP can include a global measure of psychiatric symptoms, a substance use screening, a trauma screening, an assessment of adolescent–caregiver relationship conflict, and—in keeping with the IOP’s DBT-informed approach to treatment—measures to also assess emotion regulation, distress tolerance, use of mindfulness, impulsivity, confusion about self, and interpersonal relations. FFS are designed to share the assessment data in detail with the family and provide them with information about how the results have informed treatment planning for the adolescent. FFS thus allow the family to ask for clarifications on given data and serve as a space to reflect on assessment results, allowing adolescents and their parents to review the treatment goals and make modifications.

## 9. MBC Provides Individualized Assessment and Care for Isabella and Her Family

Isabella completed the Behavior Assessment System for Children, Third Edition (BASC-3) at IOP admission. Isabella’s results were notable for elevations on multiple scales (Table 1) in the “clinically significant” range with T-scores of 70 or above, denoting severe psychiatric symptoms or problematic behaviors in the domains of Social Stress (difficulty interacting with people and trouble forming and maintaining friendships/relationships), Depression (feelings of sadness), Locus of Control (the sense that other people–e.g., parents, teachers–control one’s life), and Hyperactivity (a tendency to engage in overactive, impulsive behaviors). Isabella was “at-risk” in several areas, with lower levels of severity that may or may not intensify or require treatment. These domains included Attitude to School and Attitude to Teachers (dissatisfaction with the school environment and interactions with teachers), Sense of Inadequacy (feeling incapable of coping with life stressors and problems), Attention Problems (difficulty attending and concentrating), Anxiety (excessive worry, uneasiness, and/or fear), Relations with Parents (a negative perception of the parent–child relationship), and Interpersonal Relations (difficulty with peer relationships). Taken together, Isabella’s results indicated a high level of emotional distress.

Isabella also completed the Life Problems Inventory (LPI)–Emotion Dysregulation Scale, which measures an individual’s sensitivity and response to emotional stimuli as well as the speed at which they return to their baseline mood when their emotions become dysregulated. Higher scores reflect greater emotional sensitivity, stronger responses (e.g., suicidal thoughts/behaviors, anger outbursts), and slower return to baseline. Isabella’s score of 51 fell in the Borderline Personality Disorder (BPD) range, indicating that she likely struggled to regulate her emotions to a degree that was common for individuals who meet criteria for BPD. While it is important to note that Isabella’s score was not diagnostic of BPD, her score suggested that further evaluation would be warranted. The score also provided critical information about Isabella’s emotional functioning that could be used to assist in treatment planning.

In parallel, Isabella’s mother also completed the BASC-3, responding to questions about Isabella’s symptoms and behaviors. In contrast to Isabella’s elevated scores, her mother’s BASC-3 did not reflect any concerns, suggesting that Isabella’s mother may not have been fully aware of her internal struggles. Upon reviewing the results of these measures, the clinician was alerted to the need to use the FFS to open a channel of communication between Isabella and her parents and to provide the family with information about Isabella’s emotional struggles. The goal would be to increase their insight and understanding, and to discuss specific individual- and family-based treatment options that would be beneficial to Isabella and her family.

At the FFS, Isabella’s parents were able to gain information about her experiences of depressed mood, anxiety, feelings of inadequacy, school-related difficulties (including attention problems and hyperactivity), stress in her relations with them, and problematic relations with teachers and peers, as well as her underlying tendency toward emotional dysregulation that often manifested in anger. Isabella shared that she often struggled to calm down when angry and that she often felt misunderstood when she communicated her feelings to her parents. Isabella and her parents all agreed that they would like to work to improve communication at home and strengthen their relationships.

In light of Isabella’s assessment results, the discussion during the FFS, and elevated CBQ scores in Table 4, the clinician recommended continued family therapy as part of the aftercare plan, in conjunction with Isabella’s individual psychotherapy and medication management. Importantly, during the FFS, Isabella and her parents received education on mental health, and information on how the program process would specifically meet the family’s desired goals. This engagement incorporated the family into the treatment planning process, increasing their commitment to, and faith in, treatment.

MBC provides the opportunity for adolescents, parents, and clinicians to communicate and understand each other better during recovery. Collecting assessment data from adolescents and their family enables discrepancies to be detected and discussed [40]. This feedback-informed framework enhances clinician–patient–family engagement, therapeutic alliance, shared-decision making, and outcomes [41,42]. MBC can be applied regardless of a clinician’s or program’s theoretical orientation, and it can be used with patients who have various diagnoses [43]. However, MBC remains largely underutilized, as a national survey suggests that results of measures are shared with fewer than half of families [44]. Some underlying challenges to implement MBC include lack of engagement and time of caregivers, and family concerns around personal data being collected [45].

## 10. DBT with Multi-Family Skill Groups

For many adolescents, emotional dysregulation stems from a chronically invalidating home environment. Parents may overlook teens’ emotions and make statements like “just shake it off”, “don’t overreact”, to the detriment of their child’s emotional state. Other parents may not have appropriate parenting styles to support their adolescents’ emotional vulnerability. Founded by Marsha Linehan, DBT aims to treat chronically suicidal patients who were refractory to traditional interventions [46,47]. DBT targets four therapeutic domains: decreased self-awareness/confusion about self, impulsivity, emotion dysregulation, and interpersonal problems [47]. To address these targets, DBT skills modules include mindfulness, emotion regulation, distress tolerance, and interpersonal effectiveness. Adapting from the adult manual, DBT for Adolescents was developed to be developmentally appropriate for use with adolescents. Moreover, DBT for Adolescents includes a fifth module, “*Walking the Middle Path*”, to address adolescent–parent interpersonal conflicts via enhancing communication, understanding, problem solving and flexibility through skill building in a multi-family setting. Thus, the updated DBT skills manual provides family-system and multi-faceted approaches for a more impactful skill-building process in adolescents and their parents [48].

## 11. Isabella and Family Participate in DBT Multi-Family Skill Groups

Isabella participated in a six-week IOP (three hours a day and four days a week) with a condensed DBT IOP curriculum that covers all five of the DBT modules for adolescents. In addition, Isabella and her parents participated in virtual multi-family skill groups for one hour per week throughout the six-week IOP.

During the initial sessions, the clinician introduced the biosocial theory, which explained how emotions were experienced and how symptoms arose and were maintained [48]. For example, chronic invalidation from individuals’ environments could lead to self-invalidation and emotion dysregulation. From psychoeducation, Isabella’s mother became tearful realizing that she often told her daughter that she was overreacting or being manipulative. The multi-family skill groups also covered a module on mindfulness to increase self-awareness of emotional states, especially during conflicts. Isabella and her parents completed the mindfulness homework assignment on identifying oneself in each of the three states of mind, including Wise Mind, Reasonable Mind, and Emotional Mind. Each family member was able to acknowledge the tendency toward emotional mind. Both Isabella and her parents actively participated in mindfulness practices to help diffuse impulsive reactions while in emotional mind. The family also learned that child–parent relationships often confront dialectics—seemingly opposing ideals and feelings that must co-exist and be resolved.

At the mid-course of sessions, the family participated in a discussion on defining typical adolescent behavior vs. behavior that was the cause for concern. Isabella’s parents were able to acknowledge that they could be too strict by not allowing Isabella to spend time with friends or have a phone. Isabella also understood that she may make light of her own behaviors of self-harm and anger outbursts in ways that were unhelpful. As the family learned more about ways to validate Isabella’s feelings, the parents learned not to rush to problem solving when Isabella came to them with a problem and how this could be invalidating. The skill groups also discussed behavioral modification through reinforcement, extinction, and punishment. The family was encouraged to avoid punishment during emotionally charged interactions, as this could perpetuate Isabella’s feeling of guilt and hopelessness. As a homework assignment, Isabella and her parents co-created a list of household rules, expectations, and potential consequences.

By the end of IOP treatment, Isabella and her parents demonstrated an overall improvement in their communication and emotion co-regulation. Isabella had fewer impulsive thoughts and behaviors. These results are supported by Isabella and her parents’ reduced CBQ scores at the time of IOP discharge (Table 4).

From the DBT multi-family skill groups, families learn to validate youths’ feelings and experiences. When validation occurs in the adolescent–parent dyads, the youth’s emotional arousal reduces, self-expression becomes clearer to self and others, and tendency to engage maladaptive behaviors decreases [49]. DBT multifamily skill groups improve adolescents’ transdiagnostic problems with externalizing symptoms such as self-harm, emotional dysregulation, and impulsivity [50,51]. Multiple studies also suggest multifamily skills improve parents’ emotional well-being, distress during interactions with their adolescents, and the overall family functioning [51,52]. The multi-family setting can be a cost-effective approach in light of workforce shortages to meet rising demands of children’s mental health [53]. While it is a powerful intervention, the skill groups mainly focus on skills but may lack specificity to meet individualized family’s needs and other deep-rooted relational trauma. Moreover, overcoming resistance and promoting consistent engagement can also be a challenge. Some families may be reluctant to share in a group setting, and therefore clinicians’ facilitation and encouragement in participation will be essential.

## 12. *CHATogether*: Individualized Family Intervention

**Figure 2:**
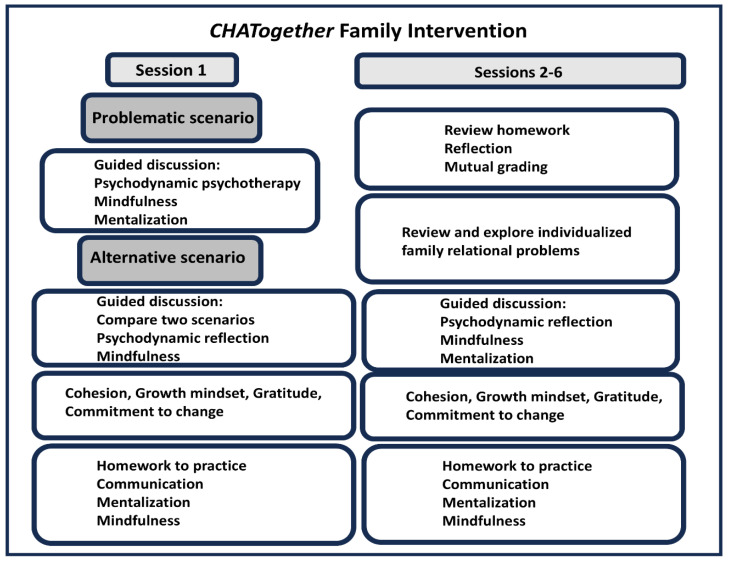
*CHATogether* family intervention protocol as part of the adolescent IOP protocol. In session 1, cinematic-guided therapy session includes problematic vs. alternative scenario with elements including psychodynamic psychotherapy, mindfulness, and mentalization. Sessions 2 to 6 include individualized family relational problems determined by individualized family questionnaires described previously [15,63]. Each session, the clinician facilitated the family with cohesion, growth mindset, gratitude, and commitment to change. Family will assign with communication exercises to complete prior to the next session. The established protocol was taken from Bookman et al., 2024 [15].

*CHATogether* is a newly developed family intervention aimed to open non-hierarchical conversations between adolescents and their parents on topics of conflict [15]. In the initial session, the family begins with a cinematic-guided discussion as their unconscious internal world is projected onto the tangible yet distant theater characters [54,55,56]. Similar to children’s play therapy and concepts of *Theatre of the Oppressed* [57,58], this approach allows psychological safety to symbolically express and process feelings and impulses that are too overwhelming or long suppressed to address directly [59,60]. In each of the subsequent sessions the families are guided to discuss topics of conflict identified prior to the session by the teen and the parents. The families are given a safe space to practice the skills of seeking middle ground, empathizing with each other, and finding sources of cohesion. Between each session, the teens and parents are asked to complete the daily listening assignment–attentively listening to each other’s day without interruption at a designated time. Individual families participate for at least 4–6 one-hour sessions throughout the adolescent’s IOP treatment. The family system approach [61,62] has been shown to target and resolve individualized family conflicts in in-person, virtual, and hybrid format (Figure 2).

## 13. *CHATogether* Teaches Isabella and Her Family to Mentalize

Mentalization is an essential reflective capacity that allows adolescents and their parents to imagine each other’s mind through explorative perspective-taking with a lens of curiosity and without judgment [64,65]. As a result, family members develop a better sense of one another’s behaviors and underlying feelings, which subsequently allows them to respond to each other’s needs sensitively and ultimately achieve affective co-regulation [66,67]. To introduce the concept of mentalization, the family watched a three-part theater skit, “*Parents got all the solutions*” (https://www.youtube.com/watch?v=5L_n4f7zbIE, accessed on 5 May 2025). The skit included the following: (1) a problematic scenario, (2) moderation, and (3) an alternative scenario as previously described [15] and in Figure 2. As the family watched the skit, and the clinician helped make drama-to-life connections and guided the family to imagine the alternative scenarios in a non-defensive manner. Throughout the sessions, each member of Isabella’s family committed to make changes in alignment with the best interests of Isabella. Facilitating cohesion, the family was asked to share their love and gratitude at the end of the session. They then went home with simple two-minute daily exercises of active listening and mentalization without interruption after each *CHATogether* session.

In the mid-course sessions, the clinicians highlight emerging relational conflicts that occurred during the recent week and allow families to practice their newfound communication skills in real time. Isabella’s mother expressed concern that when Isabella was told “no,” she “acted out” by using the word “suicide” to get others to do what she wanted. Engaging family through real-life examples, the clinician modeled the concepts of mentalization and validation, demonstrating to the family a more supportive and productive way to navigate conflicts. The clinician demonstrated possible conversing scripts for Isabella’s parents to use when Isabella becomes dysregulated and expresses suicidal ideation.

Gradually, Isabella’s parents started to recognize the degree of Isabella’s emotional suffering and began to meet her emotional needs with less judgment and criticism. Instead, they tried to take Isabella’s perspective, mentalizing her emotional pain. Isabella also gradually understood the difficulty her mother experienced trauma and violence in her earlier life, and that might be contributing to her mother’s judgmental and over-protective responses. Isabella was able to reflect that she has struggled with managing her emotions, internalized self-hatred especially when feeling “left out” by others, and often rapidly gone to extremes to “punish” herself. Her parents were able to emphasize with such pain and continue to explore the origin of this psychological pattern.

By the end of *CHATogether*, Isabella’s parents recognized that their unresolved trauma may impact on their parenting style, communication, and attunement towards Isabella’s emotions. To meet Emily’s and her family’s needs, the clinician showed a video skit titled “*Intergenerational trauma: the shark music*”. The clinician reflected on how parental trauma could play a substantial role on an adolescent’s sense of safety, self-worth, and affective regulations. Isabella and her parents treasured the opportunity to process their relational vulnerability and further commit to strengthening their relationships. Since then, Isabella’s parents were able to make co-parenting plans in the best interest of Isabella’s needs.

As shown in Table 4, Isabella and her parents showed improvement in PROMIS-depression and -anxiety after the completion of *CHATogether*. Consistently, Isabella also had a reduced CHRT-SR9 with suicide subfactors including pessimism, hopelessness, and despair [23]. As reflected by the increase in help-seeking attitude scores, Isabella showed a better acceptability to reach out to her parents and adults upon psychiatric crisis and suicide concerns. She had no change in the reject code of silence, in which she continued to hold a similar attitude that a suicidal youth should not be left alone and should seek adult help even if the youth asked her to keep it secret [24].

*CHATogether* emphasizes mutual mentalization in adolescent-parent dyads [64,65], which enhances a family’s reflective functioning for cognitive flexibility, compassionate communication, and collaborative problem solving. Drawing upon the aspects from psychodynamic therapy, drama therapy, and cognitive behavioral therapy, families develop more adaptive patterns of understanding each other’s emotions, thoughts, and behaviors as illustrated by cinematic videos [68,69]. The clinician encourages a drama-to-life translation whereby families can apply their reflections to communication patterns in daily family scenarios. Some possible limitations of this intervention may include that the cinematic videos may not be generalizable to all families. Intervention adaptations such as including storylines with diverse racial ethnics, genders, family customs, and socioeconomic status can be valuable. Through the cultivation of mindfulness [70,71], family members optimize awareness of their own emotional state, as well as their reflective and mentalization capacity [66,67]. With guided discussions and daily assignments, families can discover new ways of consciously managing experienced fears and impulses with less “fight-or-flight” attacking of self or others. *CHATogether*’s design echoes the family resilience framework where it focuses on building family relational health in helping each member adjust to stress, conflicts, and adversity [72,73].

## 14. Limitations

We concede several limitations in this case study and the overall family-centered care in the adolescent IOP. IOPs do not completely remove the adolescents from the toxic home environment that might continue to negatively affect their mental health. In Isabella’s case, the interparental arguments served as an essential stressor throughout her time in IOP. Although Isabella and her family showed clinical improvements, the case did not pinpoint which interventions are responsible for such positive outcomes. Isabella and her family did not complete all the clinical measurements offered at IOP admission and discharge. Yet, the case serves as a representative illustration of how family-based interventions can be implemented in the IOP level of care.

IOPs may not be accessible for adolescents with severe cognitive limitations, acute suicidality, or assault. Thus, there are structural barriers to which patients can receive this model of mental healthcare delivery, let alone engage in family-centered care. Another barrier to IOP implementation is the removal of adolescents from the school setting, an important context that allows for developmentally appropriate social growth and maturity [74]. Schools and employers may handle the time requirement of IOP with different levels of leniency, often leaving the onus of making up schoolwork on the individual adolescent, who is likely already behind if coming from an inpatient hospital environment. In households where academic performance are how parents define their teen’s success, this can lead to tense familial conflict. As parental engagement has been shown to be both beneficial for an adolescent’s mental health and academic success [75], the importance of utilizing the IOP to engage families in mental health will only promote greater academic success of the teen. Addressing the stigma of mental healthcare in educational settings is paramount to allow adolescents the time to properly recover from psychiatric crisis without precipitating a new academic crisis.

The family-centered care also requires a significant parental time commitment. For parents to engage in DBT multi-family skill groups, *CHATogether* family intervention, parent peer support group, and MBC FFS, they need to participate during the office hours. Although this may be feasible for families with a stay-at-home parent, the number of families with two working parents has increased from 25 to 60 percent from 1960 to 2000 [76], and continue to increase in the wake of the pandemic. Further, non-marital parenthood occurs more likely among those from lower socioeconomic backgrounds [77], and poverty rates are higher among single parent families [78]. Thus, the following question emerges: How can family-centered care in the adolescent IOP be accessible for families struggling with finances? First, many IOP programs provide transportation to and from IOPs, removing that burden from the parent and allowing the adolescent to utilize school transportation home. Another potential solution is offering virtual parental engagement opportunities, or evening sessions for parents that must remain at work throughout the day. However, over-extending parents who may already be stretched for time and financial resources are important considerations for family-centered care moving forward.

## 15. Conclusions

The IOP model serves as a vital intermediary in the delivery of care for adolescents and their families. IOP also presents a foundational venue for family engagement elements to be introduced, resulting in better outcomes. This case study illustrates family-centered care implemented during a six-week IOP structure through (1) family-centered medication management, (2) individualized case management, parental education, and peer support, (3) MBC family assessment and feedback session, (4) DBT multi-family skill groups, and (5) *CHATogether* individualized family intervention to address adolescent-parent relational health and communication. Supported by data, family-centered care shows a trend of improvement in the case’s depression/anxiety symptoms, family conflicts, suicide risks, and help-seeking behaviors. Each family component adds strength to build a stronger and cohesive family unit. The family becomes more reflective to share concerns, practice mutual understanding and communication, and ultimately work towards improving the family mental health. Although more implementation studies are warranted, such as logistic challenges for parents and program sustainability, the article introduces an essential family model in adolescent mental health treatment.

## Figures and Tables

**Table 1:** Case study demographic and admission assessments.

	Isabella	Parents
Demographic		
Age	15	
Gender	Female	
Living arrangement	Both parents	
Program participation		Both parents
Parental status		Married
Program attendance format	In-person	In-person and virtual
Diagnosis	Major Depressive Disorder PHQ-9: 16	N/A
Referral sources	Psychiatric inpatient	N/A
Admission medications	Abilify 2 mg daily Intuniv 2 mg daily	N/A
Admission psychological measures		
BASC-3	Clinical Scales:	Clinical Scales:
	Social Stress (76)	* None when compared to a clinical population.
	Depression (72)	
	Locus of Control (71)	
	Hyperactivity (71)	
	Attitude to School (69)	
	Attitude to Teachers (69)	
	Sense of Inadequacy (68)	
	Somatization (64)	
	Attention Problems (64)	
	Atypicality (62)	
	Anxiety (61)	
	Adaptive Scales:	Adaptive Scales:
	Relations with Parents (31)	* None when compared to a clinical population.
	Interpersonal Relations (32)	
LPI	51	N/A

T-scores of each domain are in parentheses. For Patient Health Questionnaire-9 (PHQ-9) modified for teens, 0–4: no depression; 5–9: mild depression; 10–14: moderate depression; 15–19: moderately severe depression; 20–27: severe depression. For Behavior Assessment System for Children 3rd Edition (BASC-3), 0–59 denotes normal range; 60–69 denotes “at risk” area of concern that may not require treatment but should be monitored; >70 suggests “clinical significant” with high level of maladjustment requiring follow-up assessment and treatment. * Scores were compared with those of other individuals identified as having behavioral and emotional difficulties. For Life Problems Inventory (LPI), <25 denotes normal range, 25–40 indicates clinical range, and ≥40 is comparable to those of individuals found to meet criteria for Borderline Personality Disorder; however, all ranges are not exact, normed, or diagnostic. N/A: Not applicable to parent participants.

**Table 2:** Example curriculum for parent peer support group.

Week	Session Topic	Goals
**1**	*CHATogether* parenting session	Promote parents’ understanding of communication skills and introduce a mentalization-based approach
**2**	Psychiatric Crisis Management	Educating parents on identifying and responding to psychiatric crises, especially regarding ensuring child safety; reducing isolation and shame among parents to demonstrate commonality of youth who experience mental health crises
**3**	Talk with a Doc: Q&A on Psychiatric medication and diagnosis	Psychoeducation on psychiatric medications and mental health diagnosis; normalization of physician-parent conversations regarding mental health; encouraging trust in medical providers among parents of children with psychiatric concerns
**4**	Motivational Interviewing for Parents: Using OARS for success	Skills training for parents on responding effectively to their child’s needs using motivational interviewing techniques of Open-Ended Questions, Affirmations, Reflections, and Summaries
**5**	Anger Management	Psychoeducation on anger and anger management techniques with accompanying skills demonstrations to assist parents in maintaining emotional regulation and model skills that they may demonstrate to encourage in their child
**6**	Mindfulness Meditation and Grounding Techniques	Psychoeducation on mindfulness and grounding techniques with accompanying skills demonstrations to assist parents in maintaining emotional regulation and model skills that they may demonstrate to encourage in their child

**Table 3:** Acceptability and feasibility of parent peer support group in 26 families including Isabella’s family.

Measures (n = 26)	% of Strongly Agree or Agree
1. Overall, I find this parent peers support group helpful for my needs	100%
2. The parent support group fits well with the existing adolescent IOP	100%
3. The parent peer support group is convenient for me as a parent to participate.	96%
4. I support the future development of parent peer support groups and recommend this service to others who may benefit from this.	100%
5. What are some other topics you would like to include in the future?	Communication with teens about substance use Teen’s self-diagnosis on internet Skills for parents to navigate limit setting Discuss safety plan in psychiatric crisis

**Table 4:** Isabella and her family’s clinical outcomes at IOP admission vs. discharge.

Measures (n = 1 from Isabella’s Case)	Isabella		Mother		Father	
	Pre	Post	Pre	Post	Pre	Post
**PROMIS-depression** (Raw/T-score)	40/61.1	23/47.6	28/60.3	22/53.5	27/59.2	11/32.1
**PROMIS-anxiety** (Raw/T-score)	38/63.1	19/45.7	25/60.4	16/45.1	29/64.8	13/38.6
**CBQ** (sum/max)	11/20	1/20	13/20	4/20	13/20	4/20
**CHRT-SR9** (sum/max) Pessimism Hopelessness Despair Suicidal Thought	18/36 6/8 6/8 6/8 0/12	0/36 0/8 0/8 0/8 0/12	N/A	N/A	N/A	N/A
**Help-seeking attitude from adults in distress and suicide concerns** (sum/max) **1. Help-seeking acceptability from parents** **2. Adult help for suicidal youth** **3. Reject codes of silence**	23/36 4/12 9/12 10/12	36/36 12/12 12/12 12/12	N/A	N/A	N/A	N/A

PROMIS T-Scores: < 55 = none to slight, 55.0–59.9 = mild, 60.0–69.9 = moderate, ≥70 = severe. PROMIS = Patient-Reported Outcomes Measurement Information System. CBQ = Conflict Behavior Questionnaires. CHRT-SR9 = Concise Health Risk Tracking Self-Report. N/A: Not applicable to parent participants.

## Data Availability

The datasets obtained and analyzed during the current study are available from the corresponding author on reasonable request.
